# Do Gender and Gender Role Orientation Make a Difference in the Link between Role Demands and Family Interference with Work for Taiwanese Workers?

**DOI:** 10.3390/ijerph18189807

**Published:** 2021-09-17

**Authors:** Luo Lu, Ting-Ting Chang, Shu-Fang Kao, Cary L. Cooper

**Affiliations:** 1 Department of Business Administration, National Taiwan University, Taipei 106, Taiwan; 2 Department of Industrial Management, Lunghwa University of Science and Technology, Guishan, Taoyuan County 33306, Taiwan; tinapc@ms24.hinet.net; 3 Department of Applied Psychology, Hsuan Chuang University, Hsinchu 30092, Taiwan; d89227002@gmail.com; 4 Alliance Manchester Business School, University of Manchester, Manchester M15 6PB, UK

**Keywords:** gender, gender role orientation, role demands, family-to-work conflict, Chinese society

## Abstract

Based on the gender role orientation perspective, this study extends the resource depletion mechanism that links role demands to family interference with work by testing the moderating effects of gender and gender role orientation (egalitarian vs. traditional) on the relationships. Analysis of the data from 251 employees in Taiwan revealed two significant three-way interactive effects. Specifically, for men, the positive relationship between work demands and family-to-work conflict (FWC) was stronger for egalitarian than traditional individuals. For women, the positive relationship between family demands and FWC was stronger for egalitarian than traditional individuals. We also found a significant two-way interactive effect; that is, within the egalitarian group, the positive relationship between work demands and FWC was stronger for women than men. Our findings, thus, suggest both within-gender and between-gender variations in the links between work-to-family demands and conflict, jointly affected by the individual’s gender and gender role orientation. Contextualized within the cultural traditions of a Chinese society, we highlight the precarious position that egalitarian men and women (especially women) find for themselves in fulfilling work duties and family roles. The theoretical and managerial implications are also discussed.

## 1. Introduction

During the year 2020, an unprecedented triple-pandemic rampaged around the entire world, involving the COVID-19 health crisis, the consequent economic recession, and the emerging social reform triggered by amplified social injustices during the crisis. In the West, lockdowns and “working from home” have blurred the demarcation between the work and home spaces, causing more excessive engagement in work activities [1]. In the East, the new virus variants have forced countries such as Taiwan to introduce draconian shutdown measures since May 2021, making “working from home” a new reality for Taiwanese workers. In the precarious post-pandemic environment, it is foreseeable that employees will be compelled to commit to more excessive work behaviors to protect job prospects [2], exposing themselves to greater risk of work and family conflict. Responding to these challenges, in the present study we aim to examine whether and how the individual’s gender and gender egalitarian attitudes will alter the linkage of role demands and work-to-family conflict. Furthermore, we aim to contribute to cultural diversity and inclusiveness in scientific research by drawing on studies of under-represented Asian populations in the literature.

Work-to-family conflict occurs when participating in one role is made more difficult by virtue of participating in the other role [3]. The mechanism of resource depletion has been widely used to explain the link between role demands and work-to-family conflict [4]. In the present study, we aim to extend the research on the resource depletion mechanism by proposing a contingency perspective. There is a tradition of exploring the role of gender in studies on work and family in the literature [5]; however, systematic reviews have shown negligible differences between men and women in terms of either the reported occurrences of work and family conflict [6] or the impacts of such conflict on role satisfaction [7]. We focus here on gender and gender role orientation and explore whether they make a difference in how individuals appraise and react to role demands in both work and family domains. Gender role orientation refers to an individual’s attitudes to how work and family roles differ based on gender in the society where they live [5,7]. Gender-related researchers have been encouraged to explore gender as a potential moderator on the work and family interface [7,8]. Moreover, because prior studies have mostly used biological sex as a proxy for gender (e.g., [6]), there is a need for more research using gender role variables (e.g., gender role orientation) that can reflect the within-gender variation [5,7]. Responding to the call, here we examine the interactive effects of gender and gender role orientation on the role demands–conflict relationships.

Our study contributes to the literature in two ways. First, according to the resource depletion mechanism, there is a positive relationship between perceived role demands and family-to-work conflict. Our study extends this mechanism by specifying how gender and gender role orientation (egalitarian vs. traditional gender attitude) moderate the relationship. Our study, thus, remedies the paucity of empirical evidence on the within-gender variation (e.g., egalitarian vs. traditional men) in the work-to-family interface, contributing to the post-pandemic “equality, diversity, and inclusion” agenda.

Second, following a cultural perspective, the context of this study is important, as the term “gender” embraces the sociocultural, psychological, and behavioral attributes associated with one’s biological sex in a particular society [7,9]. The majority of the extant literature draws on samples from Western cultures [10], with only a few exceptions (e.g., Chinese managers [11], Sri Lankan dual-careers [12], Taiwanese workers [13]). Our study, thus, explores the generalizability of the gender role orientation construct in a transitional Chinese society to advance inclusive and equitable aspects of the scientific knowledge. In contrast to the more egalitarian Western societies, traditional gender role expectations still prevail in many Eastern countries [14]. Our study, thus, extends the cultural mechanism by identifying whether and how gender role orientation (egalitarian vs. traditional gender attitude) activates different appraisal processes of role demands among male and female workers. To sum up, our findings contribute to work-to-family research by revealing whether and how gender and gender role orientation work in the process of transforming role demands into role conflict. Researchers may build upon the gender contingency perspective of the relationship between work-to-family demands and family-to-work conflict.

In the present study, we focus on family-to-work conflict (FWC) for two reasons. First, far more prior studies have focused on work-to-family conflict (WFC) than FWC [6,11]. To complement the knowledge we already have on work interfering with family, we turn to the interference from the other direction. Second, in Taiwan, where our study is contextualized, the prevailing traditional Chinese culture encourages people to view work as an essential means of fulfilling social responsibilities, and people prioritize work as instrumental for enhancing the financial welfare and social status of the family [15,16]. Thus, Chinese people are much more tolerant of work intruding into family life than their Western counterparts, as shown in cross-cultural comparisons [16,17]. Research in Taiwan has also confirmed that while the family border is highly permeable, the spillover of family matters into the work domain is not tolerated [18,19], and may incur career repercussions [20]. We, thus, focus on the moderating effects of both gender and gender role orientation on the demands–FWC relationships in the Chinese cultural context.

## 2. Theoretical Framework and Hypotheses Development

We draw on both conservation of resource (COR) and gender role theories to develop hypotheses explaining how an individual’s gender, gender role orientation, and perceived role demands *jointly* impact on work and family conflict.

### 2.1. Resource Depletion: Competing Role Demands Lead to Conflict

The basic tenet of COR theory [21] is that people strive to retain, protect, and build resources; moreover, potential or actual losses of these valued resources are stressful for individuals. As demands in work and family roles compete for the individual’s limited pool of resources, we expect that if an individual consumes resources to fulfill work demands, they then have fewer resources left to deal with family demands, and vice versa.

In order to offset the acceleration and intensification of resource depletion, people will need to conserve, mobilize, invest, and allocate resources [22]. One strategy for gaining resources is by conserving resources in one task while investing them in another. Within the context of work and family, assigning priority to the work *or* family role and reappraising role demands will enable the individual to gain and deploy more resources in the prioritized role to enhance performance [23,24]. Meta-analysis reviews have found that role saliency is related to work-to-family conflict [25,26]. Being entrenched in Chinese sociocultural prescriptions, work duties take precedence over family matters for men, whereas for women the family role is paramount; this role priority is, thus, strongly tied to one’s core self- or gender-identity and attitudes [11,16]. When facing competing role demands, men and women mobilize and invest their resources (energy, time, effort) primarily to protect the self-identified important role, which in turn enhances their sense of self-worth [15,23]. It is clear that the importance the individual assigns to a particular role dictates the decision for resource investment in role enacting. The potency of work and family roles for the individual, however, depends on both the contextual factors (i.e., role expectations of the society) and personal factors (i.e., gender role attitudes).

### 2.2. Gendered Role Expectations: The Contingency Perspective of Gender Role Orientation

Gender role theory posits that women and men are socialized to comply with prescribed gender roles and that they enact roles by exhibiting appropriate behaviors in line with the normative beliefs of the society in which they live [27,28]. Following traditional gender roles, men should put more effort into earning money and should act as breadwinners, whereas women’s proper place is in the home, meaning they should act as caregivers [27]; thus, when work-to-family demands increase, we speculate that men and women react differently according to their respective societal gender roles.

The identification of gender roles (traditional or egalitarian) motivates people to engage in self-regulation of their behaviors, which further strengthens self-consistency and underpins the management of competing role demands [23,24]. Lu, Chang, and Chang [15] found that Taiwanese workers employed different strategies to enact work-to-family roles to ensure both resource conservation and self-consistency in gender role identification. With social changes, there is now increasing within-gender variations in the identification of gender role expectations in a society [29]. For instance, “family men” (egalitarian men) coexist with breadwinners (traditional men), while “career women” (egalitarian women) coexist with homemakers (traditional women). Studies show that within-gender variation is as critical between-gender variation in explaining the differences among individuals’ experiences of work-to-family conflict [13,30,31]. We follow Livingston and Judge [8] by viewing the traditional gender role orientation as one end of a spectrum and non-traditional or egalitarian gender role orientation as the other end. Studies have found that traditional people exhibit more pronounced gender role differences in work-to-family role enacting compared with egalitarian or non-traditional people [11,13,30,31]; thus, it is important to further explore how gender role orientation makes a difference in the relationships between role demands and work-to-family conflict, both amongst men (*traditional* vs. *egalitarian men*) and amongst women (*traditional* vs. *egalitarian women*).

For *traditional men*, when work demands an increase, they will mobilize their resources to fulfill the primary role (work) obligation and spend less time and energy in family life with null or low feelings of conflict [11,32]. Similarly, *for traditional women*, when family demands increase, they will mobilize their resources to fulfill the primary role (family) or obligation and spend less time and energy on their work life with null or low feelings of conflict [15,33]. In other words, traditional men and women will prioritize the work *or* family role at the expense of the other role. This role demarcation and the subsequent resource investment strategies can be accounted for by the COR theory [22], and is consistent with their gender role identities.

The demarcation between work and family, however, is blurred for *egalitarian men and women*. *Egalitarian men* equally value work *and* family life [34]; thus, when they face increased work demands, they do not opt to sacrifice their family life [35]. While trying harder to fulfill both roles that are equally akin to their self-identities, they are more likely to overtax the resources and invoke the resource loss spiral. Such continuous resource depletion will result in stronger feelings of distress and conflict. Similarly, *egalitarian women* do not readily give up their work accomplishments as both work and family roles add to their self-worth [36]. When family demands increase, they too are more exposed to the risk of the resource loss spiral by trying harder to fulfill both roles. The differing appraisals of role importance affect the individual’s conservation and deployment of resources, resulting in varying degrees of resource depletion and strain for both traditional and egalitarian individuals. Simultaneously focusing on gender and gender role orientation, we explored *within-gender variations* to explain how the gender egalitarian attitudes of men and women influence the roles they choose to protect and how they enact them. Building on the COR theory while extending gender role orientation to a transitional society context, we hypothesized the following:

**Hypothesis** **1** **(H1).** *There will be a three-way interactive effect of gender, gender role orientation, and work demands on FWC, such that for men, gender role orientation will moderate the relationship between work demands and FWC. Specifically, the positive relationship between work demands and FWC will be stronger for egalitarian men than traditional men.*

**Hypothesis** **2** **(H2).** *There will be a three-way interactive effect of gender, gender role orientation, and family demands on FWC, such that for women, gender role orientation will moderate the relationship between family demands and FWC. Specifically, the positive relationship between family demands and FWC will be stronger for egalitarian women than traditional women.*

### 2.3. Towards Gender Equality, but Not There Yet: Gender Reality in Taiwan

As a developing and transitional society, Taiwan has a dual gender reality. On the one hand, there is a high rate of female labor participation (51.41% in 2020) and legislation protecting women’s rights and prospects at work. On the other hand, traditional gender role expectations are still influential. The results of a nationwide survey revealed that Taiwanese men as compared to women held stronger traditional gender attitudes [14]. For example, 44.3% of men endorsed a gendered role of “men as breadwinners, women as home makers” (vs. 37.1% of women). Women suffered from more barriers in advancing to managerial jobs, as well as inequality of pay and career prospects [37,38]. Women reported higher levels of work stress and strain [39]. This precarious reality is most pertinent to egalitarian women who have higher aspirations for career accomplishment and are less subjugated to traditional role expectations [40,41]. We expect that when egalitarian women exert themselves to succeed in both work and family domains, as their egalitarian male counterparts do, they face more challenges and heightened resource depletion; however, we have *no* theoretical basis for assuming that traditional men and women will differ in this respect; thus, based on COR theory and the gender reality in Taiwan, we hypothesized:

**Hypothesis** **3** **(H3).** *Within the egalitarian group, there will be a two-way interactive effect of gender and work demands on FWC, such that the positive relationship between work demands and FWC will be stronger for egalitarian women than egalitarian men.*

**Hypothesis** **4** **(H4).** *Within the egalitarian group, there will be a two-way interactive effect of gender and family demands on FWC, such that the positive relationship between family demands and FWC will be stronger for egalitarian women than egalitarian men.*

## 3. Method

### 3.1. Procedure and Participants

The participants in our study were full-time working men and women employed in different organizations of diverse industries across Taiwan. The survey was carried out using convenience sampling to recruit participants through the personal contacts of the researchers. Some participants were part-time MBA students and those enrolled in various executive education programs in universities; others were recruited through managers in various organizations. Structured questionnaires were sent out using email or hard copy. A cover letter accompanying the questionnaire informed participants of the purpose of the study and assured them of anonymity and confidentiality. Participants filled in questionnaires at their leisure and returned them in sealed envelopes to their contact persons or directly to the researchers. To encourage participation, participants were promised a small gift as a token of appreciation upon return of the completed questionnaire. One reminder was sent near the end of the set date for data collection. In total, 275 questionnaires were given out and 251 were returned (response rate: 91%), with all data being usable. The high response rate was comparable to similar studies conducted in Taiwan (e.g., [13,19]) and partly attributable to the strong social networks the researchers have built up over the years working in the field.

### 3.2. Measures

*Gender and gender role orientation.* Gender was dichotomized as men = 1 and women = 0. Gender role orientation was measured with a five-item scale that assessed the degree to which individuals endorse traditional versus egalitarian attitudes about the roles of men and women at work and home [42]. This scale was developed within the Chinese cultural context and validated in Taiwan, reflecting the gender role attitudes of the population (e.g., “Men and women should have level playing field at work”; “Men and women should share family responsibilities equally”). Participants rated each item on a 5-point scale (1 = strongly disagree, 5 = strongly agree), with higher scores representing stronger egalitarian gender role attitudes. The internal consistency of this scale was 0.82 in the present study.

*Work demands.* The quantitative workload was used to indicate work demands. Five statements from the Quantitative Workload Inventory [43] describe quantitative aspects of the work demands (e.g., “How often is there a great deal to be done?”). Respondents answered each statement by indicating the frequency of occurrence, from 1 (never happened) to 5 (always happening), with higher scores representing higher work demands. The internal consistency of the scale was 0.83 in the present study.

*Family demands.* Family responsibility was used to indicate family demands. Three statements from the Family Responsibility Scale [44] describe quantitative aspects of the family demands (e.g., “How often do you feel…that your family makes too many demands on you?”). Respondents answered each statement by indicating the frequency of occurrence, from 1 (never happened) to 5 (always happening), with higher scores representing higher family demands. The internal consistency of the scale was 0.86 in the present study.

*Family-to-work conflict.* The Work-to-Family Conflict Scale (WFCS, [45]) was used to assess FWC (e.g., “I have to put off doing things at work because of demands on my time at home”). Respondents rated the items on a five-point Likert scale (1 = absolutely incorrect, 5 = absolutely correct), with higher scores representing higher FWC. The internal consistency of the scale was 0.91 in the present study.

To exclude potential confounding factors, we controlled for age, education (in years), marital status (coded married = 1, not married = 0), number of children, living arrangement (coded living with parents/in-laws = 1, not living with parents/in-laws = 0), job tenure (in years), and managerial position (coded managers = 1, employees = 0) in all the analyses. We further controlled for work-to-family conflict (WFC, assessed by WFCS, [45]), as it has been found to moderately correlate with FWC for Taiwanese workers [13].

## 4. Results

### 4.1. Descriptive Analysis

The demographics and characteristics of the participants are summarized in Table 1. The sample was 45% male and 55% female, with a mean age of 40.35 (SD = 10.87) and mean job tenure of 8.72 years (SD = 8.11). Most participants had college-level education (with average years of education at 17.90, SD = 2.40) and over one-third of the respondents (36.70%) were managers. More employees worked in the service and manufacturing or high-tech industries (45.50%, 23.30% respectively). Over two-thirds of the sample (69.50%) were married and had children (67.50%), while 44.60% were living with parents or in-laws. To rule out any potential sampling bias, we systematically statistically controlled for the demographics and characteristics of the participants in all of the following analyses.

Prior to testing the hypotheses, bivariable correlations were computed, with the results shown in Table 2. Categorical variables (i.e., gender, marital status, living with parents, job position) were dummy-coded into 0/1 to facilitate correlational analysis. It is important to note that neither gender nor gender role orientation correlated with FWC; however, both work and family demands positively correlated with FWC.

### 4.2. Confirmatory Factor Analyses (CFA) and Testing for Common Method Variance (CMV)

To ensure that the constructs of our research model could be meaningfully distinguished, we conducted CFA to compare a series of measurement models. Specifically, our 4-factor research model where items were loaded on the theoretically assumed correlated latent factors (i.e., gender role orientation, work demands, family demands, and FWC) was set against alternative solutions (two-factor model, combining work and family demands: χ^2^/df = 4.26, CFI = 0.78, GFI = 0.77, RMSEA = 0.08, SRMR = 0.17; three-factor model, combining work demands, family demands, and FWC: χ^2^/df = 7.59, CFI = 0.51, GFI = 0.44, RMSEA = 0.11, SRMR = 0.17). The four-factor solution consistently fitted the data better (χ^2^/df = 2.29, CFI = 0.91, GFI = 0.87, RMSEA = 0.08, SRMR = 0.15), supporting the structure of our research model. As we used only the self-report measures, which may increase the threat of common method variance (CMV) bias [46], we examined an alternative model that matched our hypothesized research model, except for the inclusion of an unmeasured, latent method factor [46]. The model fit was very poor (χ^2^/df = 8.69, CFI = 0.56, GFI = 0.48, RMSEA = 0.18, SRMR = 0.18), indicating that it was unlikely that any substantial proportion of variability in responses could be attributed to the method factor.

### 4.3. Hypotheses Testing

Following Baron and Kenny’s [47] suggestion for testing and reporting moderating effects, we conducted a series of hierarchical regression analyses to test our hypotheses. Predictors were standardized and interaction terms were created from these standardized predictors. When testing H1 (work demands for men), we first entered all of the control variables in the regression model. As the second step, we entered work demands as the independent variable. As the third step, we entered gender and gender role orientation as moderators. At the fourth step, two-way interaction terms (gender × work demands, gender role orientation × work demands, gender × gender role orientation) were entered. As the final step, the three-way interaction term (gender × gender role orientation × work demands) was entered. The results are reported in Table 3.

The results in Table 3 show that having controlled for the effects of demographics, gender, gender role orientation, work demands, and two-way interactions, the focal three-way interactive effect of gender × gender role orientation × work demands on family-to-work conflict was indeed significant (Model 5); thus, Hypothesis 1 was supported.

**Figure 1:**
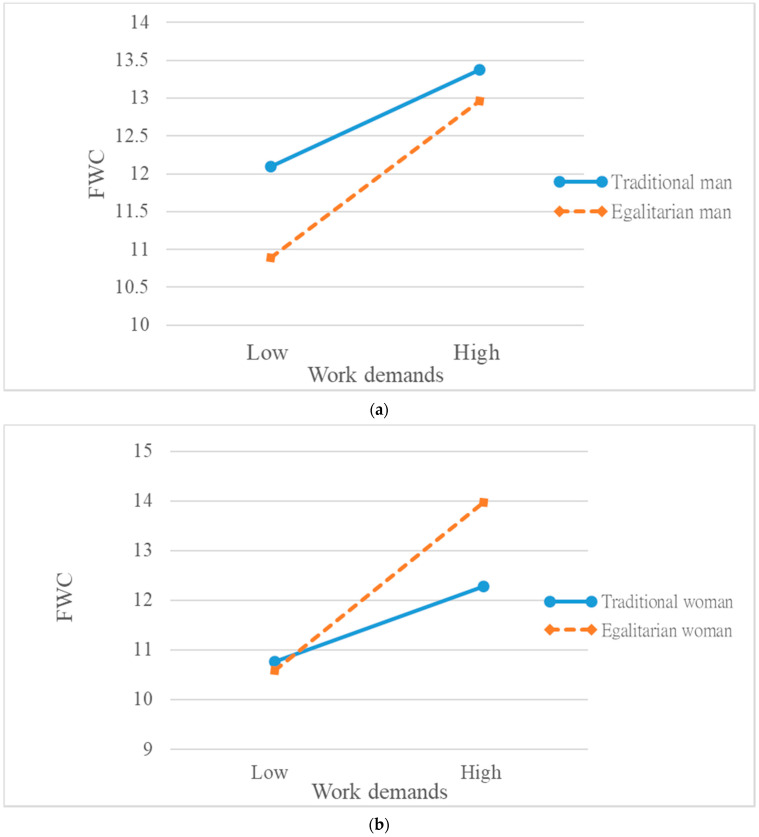
(**a**). Interactive effect of gender role orientation and work demands on family-to-work conflict (for men). (**b**) Interactive effect of gender role orientation and work demands on family-to-work conflict (for women).

As we argued earlier, for men, holding a traditional or egalitarian gender role orientation influenced their appraisals of work demands, choices of resource deployment, and subsequent feelings of conflict. Although we did not hypothesize the same within-gender difference for women, in order to present the whole picture, we nonetheless examined the nature of the three-way interaction effect both for men (Figure 1a) and women (Figure 1b). As shown in Figure 1a, for men the *positive* relationship between work demands and FWC was much *stronger* among those who endorsed a *stronger* egalitarian gender role orientation. The slopes of the two regression lines were significantly different (*t* = 1.98, *p* < 0.05). In other words, *egalitarian men* experienced *greater* FWC when work demands increased, as predicted by H1. The pattern was similar for women, as shown in Figure 1b, in the sense that the *positive* relationship between work demands and FWC was again *stronger* among those who endorsed *stronger* egalitarian gender role orientation. The slopes of the two regression lines were significantly different (*t* = 2.35, *p* < 0.01). In other words, *egalitarian women* also experienced *greater* FWC when work demands went higher. Overall, the results of this series of moderated regression analysis supported Hypothesis 1. As a *post hoc* exploration, we extended the vulnerability effect of egalitarian gender role orientation on women.

The same logic and procedure described above was followed when testing for H2 (family demands for women). The results are reported in Table 4.

The results in Table 4 showed that having controlled for the effects of demographics, gender, gender role orientation, family demands, and two-way interactions, the focal three-way interactive effect of gender, gender role orientation, and family demands on family-to-work conflict was indeed significant (Model 5); thus, Hypothesis 2 was supported.

**Figure 2:**
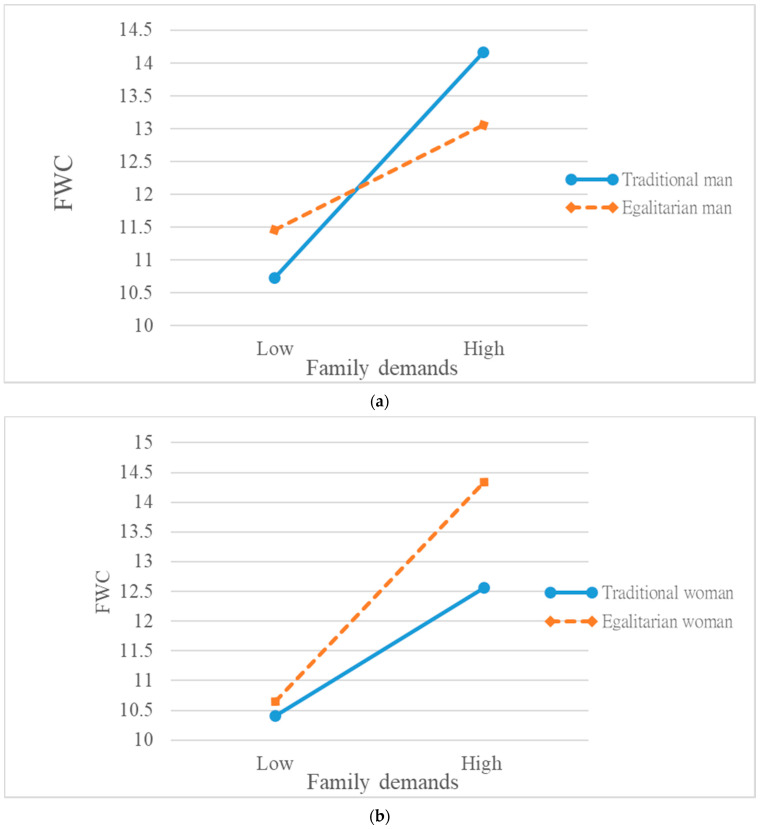
(**a**)**.** Interactive effect of gender role orientation and family demands on family-to-work conflict (for men). (**b**) Interactive effect of gender role orientation and family demands on family-to-work conflict (for women).

Again, following the same logic and procedure, we examined the nature of the three-way interactive effect both for men (Figure 2a) and women (Figure 2b). This time, we did not hypothesize the within-gender differences for men, and Figure 2a is presented only as a supplementary analysis. As shown in Figure 2b, for women, the *positive* relationship between family demands and FWC was much *stronger* among those who endorsed *stronger* egalitarian gender role orientation. The slopes of the two regression lines were significantly different (*t* = 2.03, *p* < 0.05). In other words, *egalitarian women* experienced *greater* FWC when family demands increased, as predicted by H2. An opposite pattern is shown in Figure 2a for men, in the sense that the *positive* relationship between family demands and FWC was *stronger* among those who endorsed *weaker* egalitarian gender role orientation. The slopes of the two regression lines were significantly different (*t* = 2.48, *p* < 0.01). In other words, *traditional men* experienced *greater* FWC when family demands increased. Overall, the results of this series of moderated regression analyses supported Hypothesis 2. Our *post hoc* exploration further revealed diverging effects of gender role orientation for men vs. women.

In testing for H3 and H4 (gender as a moderator on the role demands–FWC relationship within the egalitarian group), we used the median score for the gender role orientation to split the sample into the egalitarian group and the traditional group. The same protocol for moderated regression was then followed. As our hypotheses relate only to the egalitarian group, the results for this group are reported in Table 5.

The results in Table 5 show that having controlled for the effects of demographics, gender, and work demands, the two-way interactive effect of gender and work demands on family-to-work conflict was indeed significant (Model 4); thus, Hypothesis 3 was supported. The analysis for family demands, however, did not yield a significant two-way interaction of gender and family demands on FWC (Model 7); thus, Hypothesis 4 was not supported.

**Figure 3:**
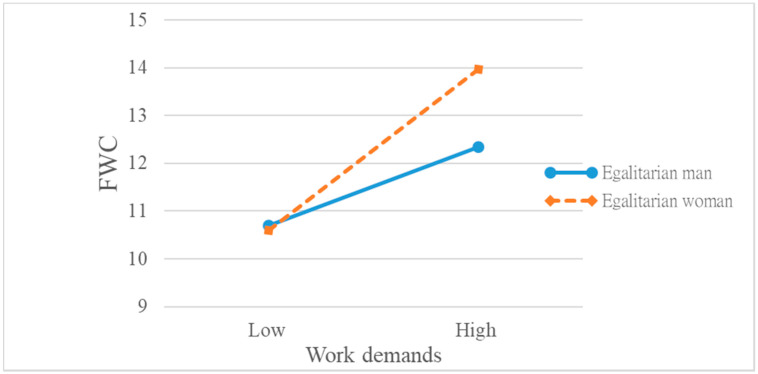
Interactive effect of gender and work demands on family-to-work conflict (for egalitarian men and women).

We again examined the nature of the two-way interactive effect for the egalitarian men and women (Figure 3). The *positive* relationship between work demands and FWC was much *stronger* for women than for men. The slopes of the two regression lines were significantly different (*t* = 2.11, *p* < 0.05). In other words, egalitarian *women* experienced *greater* FWC when work demands increased, as predicted by H3.

## 5. Discussion

### 5.1. Theoretical Implications

Our findings suggest that both between- and within-gender differences influence the mechanism of resource depletion. First, we extended the mechanism of resource depletion by identifying both gender and gender role orientation as moderators. Compared with traditional men and women, egalitarian men and women suffer more from the resource depletion caused by increasing work demands; however, when family demands increase, it is the traditional men and egalitarian women who suffer more from the resource depletion. Our findings provide the much needed empirical evidence to answer the call for focusing more on the intrapsychic gender characteristics [5,7]. Although the gender role orientation perspective theorizes that the experience of work-to-family conflict is influenced by the degree to which individuals resort to traditional versus non-traditional division of labor [8], empirical examinations of the relationship between gender role orientation and work-to-family conflict have produced mixed results. Chappell, Korabik, and McElwain [48] found no difference between those with traditional and egalitarian gender role orientations in terms of either WFC or FWC in a sample from Canada. In contrast, Ayman, Velgach, and Ishaya [49] found that egalitarian individuals experienced lower conflict than traditional individuals. These studies used gender role orientation as a *replacement* for gender. Our findings help to clarify that neither gender nor gender role orientation *per se* is sufficient to account for individual differences, it is the *joint* effect of gender and gender role orientation that makes a difference on individuals’ appraisals of role demands and their experiences of conflict; therefore, a more nuanced approach to the gender issue is required to tease out within-gender variations as people move away from the traditional gender role identity [29,50]. Our research complements the moderating effects of gender and gender role orientation found on the individual consequences of work-to-family conflict [11].

Second, while the existing work-to-family research often uses resource depletion as an explanatory mechanism, the flip side of resource conservation and investment has yet to be explored. Extending the idea that the effectiveness of resources is contingent upon the “fit” with personal and cultural values [22], we elucidate this idea by focusing primarily on the self-relevant role as a rational way of conserving resources. Traditional men and women readily identify with one role (work *or* family) and can easily concentrate their resources in one domain to maximize the fit. The dual allegiance of egalitarian men and women, however, poses a much greater challenge in terms of the effective allocation of resources. We, thus, contribute to the developing research on resource investment in the COR literature by extending it to resource allocation and utility in coping with work and family demands.

Third, situating our study within a transitional society, we contribute to the culture-sensitive perspective in developing research hypotheses [51]. Our findings highlight that the implication of gender role orientation is conditioned by the sociocultural context. The double jeopardy of being a rebel (holding egalitarian gender role orientation) and a woman is potent in the between-gender variations among the egalitarians. Despite significant social progress, traditional gender attitudes are still the dominant societal values in Taiwan [14], and the glass ceiling for women at work persists as a local reality [36]. Our findings, thus, contribute to the championing of gender equality by highlighting the vulnerability of the rebels (e.g., family men, career women) in a transitional society, especially the women moving ahead of the tide. A recent study in mainland China found that among male managers, those with an egalitarian gender role orientation suffered more negative consequences of WFC in terms of work and family accomplishment [11]. Our results extend these findings to both men and women, managers and employees. Another recent study in Taiwan [13] also revealed the vulnerability of feminine men and non-feminine women (both are viewed as non-traditional gender identities in the Chinese culture) in experiencing work-to-family conflict. In a society that puts great emphasis on conformity and fitting-in, the social pressure on the non-conformists is phenomenal [52]. Such pressure is likely to result in threats to the self, to the social image, resource depletion, and conflict over self and others’ expectations. All of these render the non-conformists stress-prone in juggling the work-to-family roles. The harsh and demanding work conditions in Taiwan make career-ambitious women more overtaxed as they are expected to demonstrate equal, if not more, work devotion, despite taking on the bulk of the family duties [39,53]. This often results in greater resource investment and depletion, such as extended working hours and reduced leisure time [36,54]. In line with encouragement of gender diversity and equity in the post-pandemic era, the plight of the high-risk groups of egalitarian men and women deserve more academic attention.

### 5.2. Managerial Implications

The stronger associations between role demands and work-to-family conflict for egalitarian men and women vis-a-vis their traditional counterparts suggest that organizations should not only adopt work–life balance measures but also actively encourage employees, especially those with egalitarian gender attitudes to make use of such resources. Organizational interventions such as flexible work hours, family-friendly practices, and supervisory support for family values have been shown to decrease work-to-family conflict [10,55]. We also found that compared with egalitarian men, women are more adversely affected by demanding job characteristics. As women already face unfair treatment at work, and often sacrifice their personal life to meet both work and family demands, such coping behaviors may incur serious negative impacts on well-being; thus, organizations should establish policies to create a level playing field for all employees to fulfill their aspirations, regardless of gender and gender role orientations.

In the post-pandemic era, various forms of flexwork (e.g., working from home, hybrid work arrangements) are challenging the traditional demarcations between the work and home [1]. People are, thus, forced to renegotiate border crossing and border management between work and non-work spaces and between on-job time and off-job time to maintain balance and satisfaction [56]. It is, thus, imperative that organizations should be fully aware of the potential downsides of work-to-life integration, such as exhaustion, reduced productivity, and diminished work–life balance [57,58]. Furthermore, organizations need to invest in training the employees’ for constructive boundary management [56,57], in order to ensure satisfaction in work (e.g., productive work performance), family (e.g., housework engagement) [57], and personal (e.g., recovery activities) [58] domains.

### 5.3. Limitations and Future Research Directions

Our study has limitations. First, as our data were cross-sectional and self-reported, causal relationships between role demands and family-to-work conflict could not be ascertained. Future researchers could adopt a longitudinal study design to establish causality. As for common method variance, our tests confirmed that all research variables were empirically distinguishable and the bias due to common method variance was low, although it would still valuable though to obtain other sources of data in future studies, such as supervisors’ or spouses’ ratings of role demands. Second, we recruited a convenience sample, which may have resulted in sample bias and restricted the robustness of our findings. Although we systematically controlled demographic and sample characteristics in all analyses, it is worth repeating our approach using large representative samples, such as nationwide surveys [14]. Third, we focused our attention on family-to-work conflict primarily because the Taiwanese society is highly tolerant of work intruding into family life but Taiwanese organizations are very intolerant of family matters spilling over into work; however, our results may not be generalizable to other cultures. Future research on cross-cultural comparisons is needed. Fourth, we adopted the resource conservation principle of COR theory to explain the differential appraisals of work and family roles for men and women, consistent with their gender role orientations. Future studies could examine what coping strategies people actually adopt in relation to their role preferences. More research is also needed to further explore other gender-related constructs, such as perceived gender equality and organizational justice, and how they may affect men and women in managing their work-to-family conflicts. Finally, Clark’s work-to-family border theory [56] is increasingly relevant in the post-pandemic “new normal” of work–non-work integration. As work–life boundary enactment strategies (e.g., segmentation, integration) have important implications for well-being, productivity, and family members’ welfare, future research needs to disentangle previous contradictory findings [57,58].

## 6. Conclusions

The present study expands on previous findings by identifying gender and gender role orientation as *joint* moderators of role demands and work-to-family conflict relationships. Our findings reveal both within-gender and between-gender variations. The initial evidence supports a culture-sensitive gendered model of role enacting and role adjustment. As our societies move towards greater gender equality, the challenges for realizing both the work and family aspirations of egalitarian individuals, especially egalitarian women, deserve more research attention and organizational intervention. In the post-pandemic world, a truly equitable and inclusive understanding of men’s and women’s lived experiences in work-to-family roles will only be possible when researchers take into account the full biopsychosocial implications of gender.

## Figures and Tables

**Table 1:** The descriptive statistics for the ample (N = 251).

Variables	N	Valid Percent (%)	Mean (Range)	Standard Deviation
**Gender**	249			
Male	112	45		
Female	137	55		
Missing	2			
**Age**	249		40.35 (19–70)	10.87
Missing	2			
**Job tenure**	246		8.72 (0.80–42)	8.11
Missing	5			
**Marital status**	249			
Married or cohabiting	173	69.50		
Single	60	24.10		
Widowed, separated, or divorced	16	6.40		
Missing	2			
**Job position**	248			
Top-level executive	16	6.50		
Middle-level manager	37	14.90		
Lower-level manager	38	15.30		
Non-manager	157	63.30		
Missing	3			
**Education attainment**	232			
High school/vocational school	84	36.20		
College/university	114	49.20		
Graduate school	34	14.60		
Missing	19			
**Education years**	232		17.90 (15–25)	2.40
missing	19			
**Industrial classification**	249			
Manufacturing/High-tech	58	23.30		
Service	113	45.50		
Civil service/Education	25	10.00		
Financial industry	25	10.00		
Retail/trade	10	4.00		
Other	18	7.20		
Missing	2			
**Children living together**	249			
Yes	168	67.50		
No	81	32.50		
Missing	2			
**Living with parents or in-laws**	249			
Yes	111	44.60		
No	138	55.40		
Missing	2			

*Notes:* Education years: high school/vocational school = 15 years; college/university = 19 years; graduate school = 21 years.

**Table 2:** Interrelations among research variables (N = 251).

		Mean	SD	1.	2.	3.	4.	5.	6.	7.	8.	9.	10.	11.	12.
1.	Gender	0.55	0.50												
2.	Age	39.81	10.75	−0.10											
3.	Education(y)	17.91	2.41	−0.06	−0.21 **										
4.	Maritalstatus	0.68	0.47	−0.05	0.50 ***	−0.14 *									
5.	#Children	0.64	0.48	0.03	0.63 ***	−0.24 ***	0.69 ***								
6.	Livingwithparents	0.45	0.50	−0.02	−0.32 ***	0.03	−0.23 **	−0.33 ***							
7.	Jobtenure	8.29	7.69	−0.09	0.57 ***	−0.10	0.27 ***	0.34 ***	−0.10						
8.	Jobposition	0.36	0.48	−0.27 ***	0.10	0.11	0.07	0.05	0.09	0.28 ***					
9.	Workdemands	17.14	3.25	0.13 *	−0.04	0.02	0.01	0.00	0.00	0.05	0.11	(0.83)			
10.	Familydemands	8.13	2.47	0.07	0.11	−0.09	0.20 **	0.26 ***	−0.04	0.18 **	0.00	0.12	(0.86)		
11.	FWC	12.04	3.99	−0.03	−0.02	−0.11	0.00	0.00	−0.10	0.03	0.00	0.10	0.25 ***	(0.91)	
12.	Genderroleorientation	24.60	3.45	0.42 ***	−0.03	−0.05	−0.06	0.00	−0.10	−0.10	−0.10	0.26 ***	−0.09	0.00	(0.82)

Notes: (1) Scale reliabilities (Cronbach’s alphas) appear in brackets on the diagonal. Partial correlation coefficients are shown for FWC controlling for WFC. (2) Gender: 0 = women; 1 = men; Marital status: 0 = not married; 1 = married. Living with parents: 0 = no; 1 = yes. Job position: 0 = employees; 1 = managers. FWC = family-to-work conflict. (3) Note: * *p* < 0.05, ** *p* < 0.01, *** *p* < 0.001.

**Table 3:** Interactive effects of gender, gender role orientation, and work demands on family-to-work conflict (N = 251).

Variables	Model 1	Model 2	Model 3	Model 4	Model 5
Control Variables					
Age	−0.21 *	−0.14	−0.14	−0.15	−0.12
Education (y)	−0.11	−0.10	−0.11	−0.09	−0.08
Marital status	0.02	0.00	−0.02	−0.02	−0.02
# Children	0.04	0.02	0.04	0.04	0.01
Living with parents	−0.17 *	−0.14 *	−0.15 *	−0.14 *	−0.15
Job tenure	0.08	0.05	0.04	0.03	0.03
Job position	0.12	0.07	0.03	0.04	0.03
Independent variable					
Work demands		0.32 ***	0.35 ***	0.24 *	0.22 *
Moderators					
Gender			−0.08	−0.08	−0.09
Gender role orientation			−0.09	−0.19	−0.19*
Two-way interactions					
Gender × Gender role orientation				0.12	0.11
Gender × Work demands				0.15	0.10
Gender role orientation × Work demands				0.00	−0.13
Three-way interaction					
Gender × Gender role orientation × Work demands					0.22 *
Adjusted R^2^	0.06	0.15	0.17	0.19	0.21
∆R^2^	0.06	0.10 ***	0.02	0.02	0.02 *
F	1.82	4.96 ***	4.53 ***	3.92 ***	4.09 ***
∆F	1.82	25.53 ***	2.54	1.71	5.29 *
(df)	(7, 219)	(8, 218)	(10, 216)	(13, 213)	(14, 212)

*Notes:* (1) All coefficients are standardized beta coefficients. (2) Gender: 0 = female; 1 = male. Marital status: 0 = not married; 1 = married. Living with parents: 0 = no; 1 = yes. Job position: 0 = employees; 1 = managers. (3) Note: * *p <* 0.05, *** *p <* 0.001.

**Table 4:** Interactive effects of gender, gender role orientation, and family demands on family-to-work conflict (N = 251).

Variables	Model 1	Model 2	Model 3	Model 4	Model 5
Control variables					
Age	−0.21 *	−0.11	−0.13	−0.12	−0.12
Education (y)	−0.11	−0.10	−0.10	−0.08	−0.07
Marital status	0.02	−0.01	−0.01	−0.01	0.00
# Children	0.04	−0.07	−0.06	−0.07	−0.08
Living with parents	−0.17 *	−0.17 *	−0.17 *	−0.16 *	−0.16 *
Job tenure	0.08	0.01	0.02	0.01	0.00
Job position	0.12	0.12	0.09	0.10	0.11
Independent variable					
Family demands		0.38 ***	0.39 ***	0.36 **	0.34 **
Moderators					
Gender			−0.10	−0.10	−0.06
Gender role orientation			0.03	−0.09	−0.10
Two-way interactions					
Gender × Gender role orientation				0.15	0.15
Gender × Family demands				0.03	−0.02
Gender role orientation × Family demands				0.00	−0.15
Three-way interaction					
Gender × Gender role orientation × Family demands					0.23 *
Adjusted R^2^	0.06	0.19	0.19	0.20	0.22
∆R^2^	0.06	0.13 ***	0.01	0.01	0.01 *
F	1.87	6.25 ***	5.22 ***	4.20 ***	4.21 ***
∆F	1.87	34.87 ***	1.09	0.84	3.70 *
(df)	(7, 220)	(8, 219)	(10, 217)	(13, 214)	(14, 213)

*Notes:* (1) All coefficients are standardized beta coefficients. (2) Gender: 0 = female; 1 = male. Marital status: 0 = not married; 1 = married. Living with parents: 0 = no; 1 = yes. Job position: 0 = employees; 1 = managers. (3) Note: * *p <* 0.05, ** *p <* 0.01, *** *p <* 0.001.

**Table 5:** Interactive effects of gender and work and family demands on family-to-work conflict among egalitarian men and women (N = 102).

Variables	Model 1	Model 2	Model 3	Model 4
Control variables				
Age	−0.16	−0.02	−0.03	0.00
Education (y)	−0.11	−0.01	−0.01	0.01
Marital status	−0.05	−0.16	−0.16	−0.17
# Children	0.12	−0.01	0.01	−0.02
Living with parents	−0.18	−0.11	−0.10	−0.09
Job tenure	−0.02	−0.13	−0.13	−0.13
Job position	0.14	0.05	0.04	0.02
Independent variables				
Work demands		0.28 **	0.28 **	0.05
Family demands		0.39 **	0.40 **	0.45
Moderator				
Gender			−0.04	−0.08
Interactions				
Gender × Work demands				0.31 *
Gender × Family demands				−0.07
Adjusted R^2^	0.05	0.28	0.28	0.32
∆R^2^	0.05	0.23 ***	0.00	0.03
F	0.71	3.76 **	3.36 **	3.22 **
∆F	0.71	13.70 ***	0.14	2.06
(df)	(7, 88)	(9, 86)	(10, 85)	(12, 83)

*Notes:* (1) All coefficients are standardized beta coefficients. (2) Gender: 0 = female; 1 = male. Marital status: 0 = not married; 1 = married. Living with parents: 0 = no; 1 = yes. Job position: 0 = employees; 1 = managers. (3) Note: * *p <* 0.05, ** *p <* 0.01, *** *p* < 0.001.

## Data Availability

The data that support the findings of this study are available from the corresponding author, L.L., upon reasonable request.
